# Compositions of fungal secretomes indicate a greater impact of phylogenetic history than lifestyle adaptation

**DOI:** 10.1186/1471-2164-15-722

**Published:** 2014-08-27

**Authors:** Jorrit-Jan Krijger, Michael R Thon, Holger B Deising, Stefan GR Wirsel

**Affiliations:** Institut für Agrar- und Ernährungswissenschaften, Naturwissenschaftliche Fakultät III, Martin-Luther-Universität Halle-Wittenberg, Betty-Heimann-Str. 3, Halle, (Saale) D-06120 Germany; Centro Hispano-Luso de Investigaciones Agrarias, Universidad de Salamanca / Parque Científico, Campus de Villamayor, C/ Río Duero, 12, Villamayor, (Salamanca) 37185 Spain; Interdisziplinäres Zentrum für Nutzpflanzenforschung, Martin-Luther-Universität Halle-Wittenberg, Betty-Heimann-Str. 3, Halle, (Saale) D-06120 Germany

**Keywords:** Fungi, Secretome, Cysteine-rich, SSP, SSCP, Cluster, Contraction, Expansion, Phylogeny, Nutritional lifestyle

## Abstract

**Background:**

Since the first fungal genome sequences became available, investigators have been employing comparative genomics to understand how fungi have evolved to occupy diverse ecological niches. The secretome, i.e. the entirety of all proteins secreted by an organism, is of particular importance, as by these proteins fungi acquire nutrients and communicate with their surroundings.

**Results:**

It is generally assumed that fungi with similar nutritional lifestyles have similar secretome compositions. In this study, we test this hypothesis by annotating and comparing the soluble secretomes, defined as the sets of proteins containing classical signal peptides but lacking transmembrane domains of fungi representing a broad diversity of nutritional lifestyles. Secretome size correlates with phylogeny and to a lesser extent with lifestyle. Plant pathogens and saprophytes have larger secretomes than animal pathogens. Small secreted cysteine-rich proteins (SSCPs), which may comprise many effectors important for the interaction of plant pathogens with their hosts, are defined here to have a mature length of ≤ 300 aa residues, at least four cysteines, and a total cysteine content of ≥5%. SSCPs are found enriched in the secretomes of the Pezizomycotina and Basidiomycota in comparison to Saccharomycotina. Relative SSCP content is noticeably higher in plant pathogens than in animal pathogens, while saprophytes were in between and closer to plant pathogens. Expansions and contractions of gene families and in the number of occurrences of functional domains are largely lineage specific, e.g. contraction of glycoside hydrolases in Saccharomycotina, and are only weakly correlated with lifestyle. However, within a given lifestyle a few general trends exist, such as the expansion of secreted family M14 metallopeptidases and chitin-binding proteins in plant pathogenic Pezizomycotina.

**Conclusions:**

While the secretomes of fungi with similar lifestyles share certain characteristics, the expansion and contraction of gene families is largely lineage specific, and not shared among all fungi of a given lifestyle.

**Electronic supplementary material:**

The online version of this article (doi:10.1186/1471-2164-15-722) contains supplementary material, which is available to authorized users.

## Background

Secreted proteins are essential for interactions with the environment, particularly for sessile organisms such as fungi. Fungi exhibit a wide diversity of nutritional lifestyles, ranging from strict saprobes, living only of dead organic material, through necrotrophic pathogens to obligate biotrophic mutualists and pathogens, existing within the living tissue of a host. Accordingly, fungal secreted proteins play important roles in the degradation of complex organic compounds, and in modulating directly or indirectly symbiotic and pathogenic interactions with their hosts. Effectors are secreted proteins of plant and animal pathogens, which are involved in manipulating, directly or indirectly, defense reactions and metabolism of their hosts [1–3]. For example, the maize pathogen *Ustilago maydis* secretes a chorismate mutase that is translocated into infected maize cells to manipulate the shikimate pathway, which, amongst others, is involved in the production of salicylate, an important signal eliciting host defenses such as the systemic acquired resistance (SAR) [4]. Other effectors prevent the detection of pathogen-associated molecular patterns (PAMPs), which are structurally conserved microbial molecules, by specific receptors of the host [5, 6]. For instance, chitin is an important structural component of fungal cell walls but also acts as a PAMP. However, in compatible plant-pathogen interactions, chitin does not elicit effective host defenses as it is either modified by extracellular deacetylation to chitosan or shielded by chitin-binding effectors [7–9]. Thus, secreted proteins play essential roles in infection and determine whether a fungus may be an effective pathogen for a given host.

Most of the fungal secreted proteins are believed to be transported through the type II secretion pathway [10]. Proteins that are destined for this pathway contain an N-terminal signal peptide that mediates recognition and transport across the endoplasmic reticulum (ER) membrane by a membrane-embedded translocon. The signal peptide is cleaved off and is thus not part of the mature protein. During transport through the ER and the Golgi apparatus the protein is folded, in many cases glycosylated and then travels from the Golgi to one of several possible final destinations, one of which is the extracellular space [11–15].

Secreted proteins have been identified by molecular genetic and biochemical methods, for example the yeast signal sequence trap [16], but these typically identify only a fraction of the presumed total secretome. The signal peptide that is characteristic of the type II secretion pathway is highly conserved among eukaryotes, which allowed the development of computer programs such as SignalP [17], TargetP [18], and Phobius [19] for its identification in protein sequences. This, coupled with the increasing availability of whole genome sequences for fungi, provides us with the resources to identify computationally the predicted set of secreted proteins (the secretome) in a large number of fungi representing various nutritional lifestyles and phylogenetic lineages.

Previous studies of fungal secretomes suggested that the composition of gene families varies greatly among species, and that some gene families appear to be associated with certain phylogenetic lineages and/or lifestyles [20]. Using gene model annotations from genome sequences, we performed a computational identification of soluble, secreted proteins, i.e. proteins containing a signal peptide, but no signal anchor or transmembrane domains, in species representing both a broad phylogenetic diversity as well as diversity in lifestyles. This allows assessing critically the hypothesis that species sharing a nutritional lifestyle have secretomes that exhibit a similar composition. In this context, we specifically address the questions (1) whether secretome size and composition correlates with lifestyle and/or with phylogenetic lineages, (2) whether such observed differences might be the result of expansion or contraction of certain gene families, and (3) whether altered occurrences of functional units/domains, independent of gene families, might explain the differences.

## Results

### Prediction and characterization of fungal secretomes

The annotated gene models for 33 fungal and three phytopathogenic Oomycota genomes were obtained from various databases (Table 1, Additional file 1). The fungal collection comprised 21 Ascomycota, eight Basidiomycota, two Zygomycota, one Chytridiomycota, and one Microsporidium. The three Oomycota genomes were included as outgroup for the plant pathogens within the Mycota. The selected fungal species did not only cover all phyla of the Mycota but also represented a wide range of nutritional lifestyles. Thus, 15 species are saprophytes, eight are plant pathogens including one obligate biotroph, eight are vertebrate pathogens (7 mammalian pathogens plus the amphibian pathogen *Batrachochytrium dendrobatidis*), one is an ectomycorrhizal, and one is a polyphagous fungus (Table 1, Additional file 1). This latter species, i.e. *Rhizopus oryzae* [21], has been found in association with plants but is also described as a mammalian pathogen and a saprophyte.

**Table 1:** **Species analyzed**

Name	Code	Lifestyle	Phylum	Subphylum
*Ashbya gossypii*	Agos	Plant pathogen	Ascomycota	Saccharomycotina
*Aspergillus fumigatus*	Afum	Animal pathogen	Ascomycota	Pezizomycotina
*Aspergillus nidulans*	Anid	Saprophyte	Ascomycota	Pezizomycotina
*Batrachochytrium dendrobatidis*	Bden	Animal pathogen	Chytridiomycota	n.a.
*Botrytis cinerea*	Bcin	Plant pathogen	Ascomycota	Pezizomycotina
*Candida albicans*	Calb	Animal pathogen	Ascomycota	Saccharomycotina
*Candida glabrata*	Cgla	Animal pathogen	Ascomycota	Saccharomycotina
*Candida lusitaniae*	Clus	Animal pathogen	Ascomycota	Saccharomycotina
*Chaetomium globosum*	Cglo	Saprophyte	Ascomycota	Pezizomycotina
*Coccidioides immitis*	Cimm	Animal pathogen	Ascomycota	Pezizomycotina
*Coprinus cinereus*	Ccin	Saprophyte	Basidiomycota	Agaricomycotina
*Cryptococcus neoformans*	Cneo	Animal pathogen	Basidiomycota	Agaricomycotina
*Debaryomyces hansenii*	Dhan	Saprophyte	Ascomycota	Saccharomycotina
*Encephalitozoon cuniculi*	Ecun	Animal pathogen	Microsporidia	n.a.
*Fusarium graminearum*	Fgra	Plant pathogen	Ascomycota	Pezizomycotina
*Kluyveromyces lactis*	Klac	Saprophyte	Ascomycota	Saccharomycotina
*Laccaria bicolor*	Lbic	EcM	Basidiomycota	Agaricomycotina
*Magnaporthe oryzae*	Mory	Plant pathogen	Ascomycota	Pezizomycotina
*Neurospora crassa*	Ncra	Saprophyte	Ascomycota	Pezizomycotina
*Phanerochaete chrysosporium*	Pchr	Saprophyte	Basidiomycota	Agaricomycotina
*Phycomyces blakesleeanus*	Pbla	Saprophyte	Zygomycota	Mucoromycotina
*Phytophthora infestans*	Pinf	Plant pathogen	Oomycota	n.a.
*Phytophthora ramorum*	Pram	Plant pathogen	Oomycota	n.a.
*Phytophthora sojae*	Psoj	Plant pathogen	Oomycota	n.a.
*Pichia stipitis*	Psti	Saprophyte	Ascomycota	Saccharomycotina
*Postia placenta*	Ppla	Saprophyte	Basidiomycota	Agaricomycotina
*Puccinia graminis*	Pgra	Plant pathogen	Basidiomycota	Pucciniomycotina
*Rhizopus oryzae*	Rory	Polyphage	Zygomycota	Mucoromycotina
*Saccharomyces cerevisiae*	Scer	Saprophyte	Ascomycota	Saccharomycotina
*Schizosaccharomyces pombe*	Spom	Saprophyte	Ascomycota	Taphrinomycotina
*Sclerotinia sclerotiorum*	Sscl	Plant pathogen	Ascomycota	Pezizomycotina
*Sporobolomyces roseus*	Sros	Saprophyte	Basidiomycota	Pucciniomycotina
*Stagonospora nodorum*	Snod	Plant pathogen	Ascomycota	Pezizomycotina
*Trichoderma reesei*	Tree	Saprophyte	Ascomycota	Pezizomycotina
*Ustilago maydis*	Umay	Plant pathogen	Basidiomycota	Ustilaginomycotina
*Yarrowia lipolytica*	Ylip	Saprophyte	Ascomycota	Saccharomycotina

Names, codes used in figures, lifestyles, phyla, and subphyla of the species analyzed in this study, ordered alphabetically.

**Figure 1:**
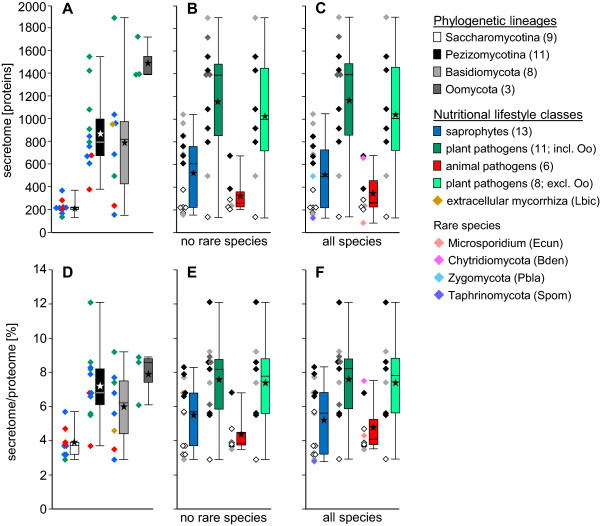
**Secretome sizes in relation to phylogeny and lifestyle.** Absolute **(A - C)** and relative **(D - F)** sizes of secretomes. **A**, **B**, **D**, **E**: species from phyla with at least three members. Rare species: the only representative of a phylogenetic lineage in a nutritional lifestyle group. **C**, **F**: all species sharing a nutritional lifestyle except *L. bicolor*, as it is a symbiont, and *R. oryzae*, as it is polyphagous. **A**, **D**: species grouped by phylogeny. **B**, **C**, **E**, **F**: species grouped by nutritional lifestyle. Diamonds: data points, colored according to classification opposite to boxes. Boxes: lower and upper quartiles. Internal line: median. Lower and upper whiskers: minimum and maximum. Asterisks: means. Greyscales: phylogenetic groups. Colors: lifestyle groups.

Proteins containing a type II secretion signal sequence were identified using the computer programs SignalP 2.0 and 3.0, and TargetP [17, 18]. A protein was only considered secreted when all three algorithms predicted this feature, according to [22], which yielded an average of 10.4% signal peptide-containing proteins per species (Additional file 1). As we focused our study on proteins that are soluble, we subtracted all proteins carrying transmembrane domains from this initial set. These domains were identified by the program Phobius that was specifically designed to distinguish between N-terminal transmembrane domains (TMs) and “classical” type II secretion signal sequences [23]. This showed that the soluble secretome constituted on average 5.8% of the proteome with extreme values of 2.8% for *Schizosaccharomyces pombe* and 12.1% for *Magnaporthe oryzae* (Additional file 1). When assessing these soluble secretomes in a phylogenetic context, Pezizomycotina and Basidiomycota had similar sizes, whereas the average size of Saccharomycotina was noticeably smaller (Figure 1). Comparing the soluble secretomes of fungi differing in nutritional lifestyle, saprophytes and animal pathogens had similar sizes, whereas the secretomes of plant pathogens were larger. These differences held true for both absolute (Figure 1 A-C) and relative size (Figure 1 D-F). At the level of absolute size of the soluble secretomes, the 36 species were grouped into three classes. Class 1 contained 14 species with secretomes comprising less than 400 proteins (Additional file 1). Class 2 comprised 16 species with secretomes ranging from ca. 500 to 1100 proteins. Class 3 included the remaining six species with secretomes between ca. 1400 and 1900 proteins. In parallel, however, the species represented in these absolute secretome size classes also have increasingly large proteomes (Additional file 1), which shows relative secretome size to be the more valid comparator for our data set.

Comparing relative sizes of the soluble secretomes with phylogeny, within the Ascomycota the Saccharomycotina and Taphrinomycotina have secretomes in the range of 2.8% to 5.7% of the proteomes with an average of 3.8%, whereas the Pezizomycotina secretomes range from 3.7% to 12.1% of the proteomes with an average of 7.3% (Figure 1D, Additional file 1). The Basidiomycota secretomes cover a broad size range from 2.9% to 9.2% of the proteomes, with an average of 6.0%. Overall, Ascomycota have relative secretome sizes from 2.8% to 12.1% with an average of 5.6% and thus cover a similar size range as the Basidiomycota, with unicellular species at the lower and filamentous species at the higher ends (Figure 1D, Additional file 1).

**Figure 2:**
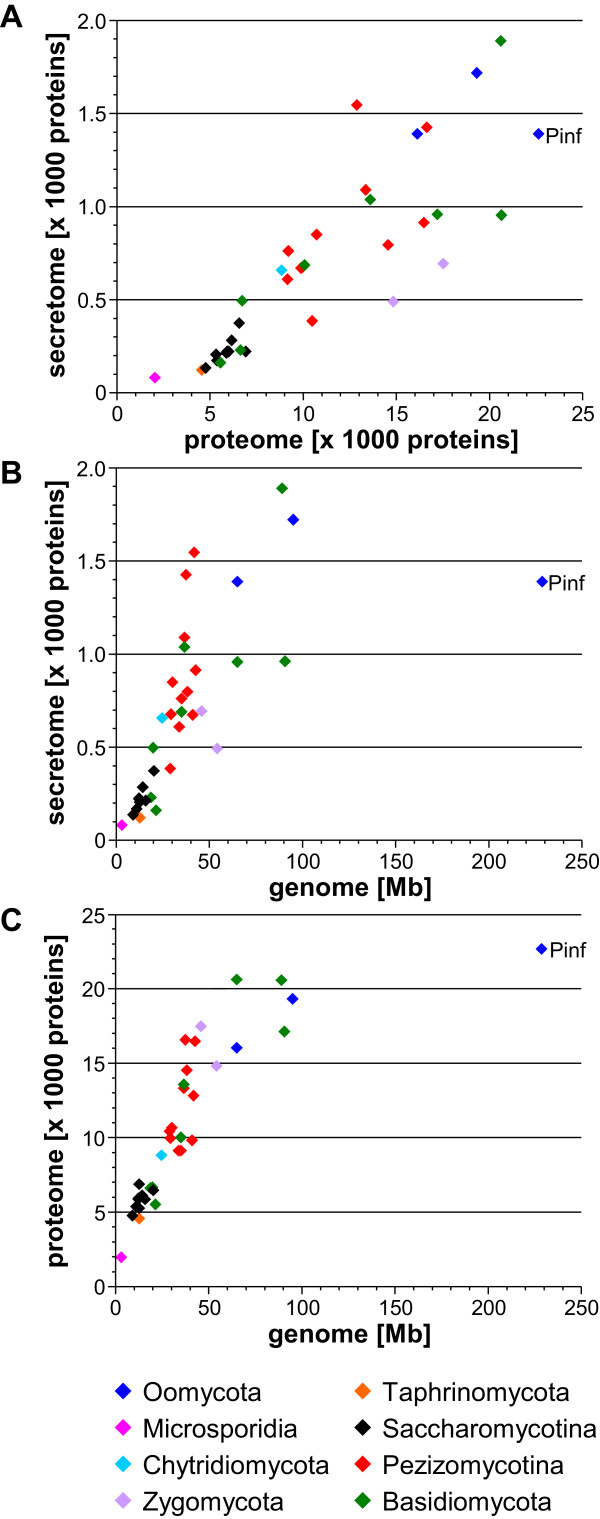
**Correlations between the absolute sizes of genomes, proteomes, and soluble secretomes. (A)** Absolute sizes of predicted soluble secretomes vs. predicted proteomes. **(B)** Absolute sizes of predicted soluble secretomes vs. genomes. **(C)** Absolute sizes of predicted proteomes vs. genomes. Data points are color-coded by phylogeny. Abbreviations for species are given in Table 1.

In relation to nutritional lifestyle, animal pathogen secretomes range from 3.5% to 7.5% of the proteomes with an average of 4.4%, whereas plant pathogen secretomes range from 2.9% to 12.1% of the proteomes with an average of 7.4% and thus are considerably larger. The sizes of the secretomes of saprophytes range from 2.8% to 8.3% with an average of 5.2% (Figures 1E, F, Additional file 1). When comparing, in Dikarya, the 13 unicellular or dimorphic yeasts with the 16 filamentous fungi (the species with genomes smaller than 15 Mb and proteomes smaller than 7000 proteins versus the species with genomes larger than 44 Mb and proteomes of more than 9000 proteins), it is obvious that the former group of taxa typically do not only have small absolute but also small relative secretomes. The large-proteome filamentous fungi, on the other hand, rather have large secretomes both absolutely and relatively (Additional file 1). The plot of secretome size versus proteome size shows the general trend of increase of the former with increase of the latter (Figure 2A). The similar, small secretome-to-proteome size ratios of the Saccharomycotina are clearly seen in the form of a “dip” of the data points in the lower left corner of the graph, while for the other species, these show more variation. Plots of secretome size vs. genome size (Figure 2B) and proteome size vs. genome size (Figure 2C) show similar, near-linear relationships with increasing variability for the larger genomes, secretomes, and proteomes. In the latter two comparisons, *Phytophthora infestans* appears as an extreme case, which is based in the massive expansion of its genome, due to increased numbers of repetitive elements [24].

**Figure 3:**
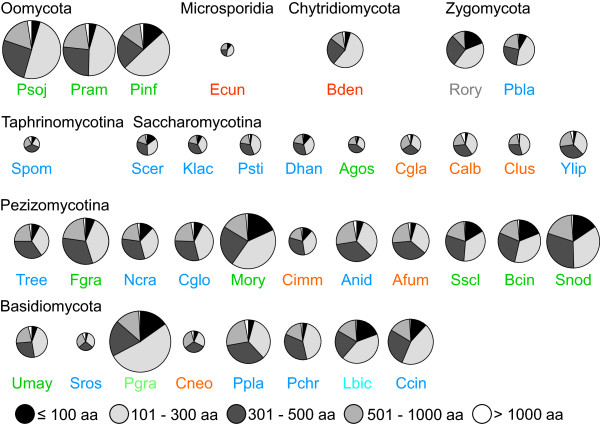
**Protein size distributions within soluble secretomes.** Number of proteins in five length intervals (≤100 aa, 101-300 aa, 301-500 aa, 501-1000 aa and >1000 aa) as fractions of the total soluble secretomes. Circle areas correspond to the absolute sizes of the soluble secretomes. Species denominators are color-coded by lifestyle: dark blue = saprophytes, dark green = plant pathogens, red = animal pathogens, grey = polyphage (*R. oryzae*), light blue = Ectomycorrhiza (EcM, *L. bicolor*), light green = obligate biotroph (*P. graminis*). Abbreviations for species are given in Table 1.

For each secretome, protein length distribution was analyzed by depicting the numbers of proteins in defined length intervals (≤100, 101-300, 301-500, 501-1000 and ≥1001 aa) as fractions of the total secretome (Figure 3). The majority of proteins in each secretome had less than 500 aa (from 62.3% in *Sporobolomyces roseus* to 86.4% in *R. oryzae*). However, proteins of up to 100 aa residues were remarkably enriched in four of the five plant pathogenic Pezizomycotina analyzed, i.e. *Botrytis cinerea*, *M. oryzae*, *Stagonospora nodorum,* and *Sclerotinia sclerotiorum*, making up 10% to 15% of the total secretomes. A similar enrichment of such small proteins was further observed only in the Ectomycorrhiza (EcM) fungus *Laccaria bicolor*, in the obligate biotrophic plant pathogen *Puccinia graminis*, both belonging to the Basidiomycota, and in the polyphagous Zygomycete *R. oryzae*.

We further divided the secretomes based on cysteine content and size since previous work indicated that putative effectors are often small cysteine-rich proteins [25–27]. Small secreted proteins (SSPs) are defined here to have a mature length of ≤ 300 aa residues. If such proteins had a relative cysteine content of ≥5%, as well as ≥4 cysteine residues, we considered them as small secreted cysteine-rich proteins (SSCPs, Table 2A, Additional file 2A). Using these criteria, we found that cysteine-rich proteins were overall clearly enriched in the SSPs (Table 2, compare SSCPs to C-rich total), which was not obvious when applying a more relaxed definition for cys-enrichment using only a minimum of four residues but not considering the percentage of cysteine residues (Table 2B, Additional file 2B). Class 2 and 3 size secretomes had more than two-fold and three-fold higher SSCP-to-secretome ratios than the small class 1 size secretomes, respectively, suggesting that the increase in secretome size may connect to a relative increase of SSCP content. Pezizomycotina and Basidiomycota had close to four-fold higher SSCP-to-secretome ratios than Saccharomycotina, and notably higher ratios than Oomycota. Plant pathogens had a two-and-a-half-fold higher SSCP-to-secretome ratio than animal pathogens, while saprophytes were in between and closer to plant pathogens (Additional file 2A).

**Table 2:** **Relative cysteine contents by secretome size, phylogeny and lifestyle**

A) Stringent definition: cysteine-rich ≥4 cysteines and ≥5% cysteines				
	Species	C-rich total (%)	SSPs (%)	SSCPs (%)
Overall	36	6.3	47	5.5
Secretome size				
Class 1	14	3.2	41	2.8
Class 2	16	7.6	49	6.7
Class 3	6	9.6	57	8.6
Phylogeny				
Oomycota	3	6.1	56	4.7
Saccharomycotina	9	2.2	42	2.1
Pezizomycotina	11	8.6	47	7.5
Basidiomycota	8	8.6	48	8.0
Lifestyle				
Saprophyte	15	5.9	43	5.4
Plant pathogen	11	8.0	52	7.0
Animal pathogen	8	3.7	44	2.8
**B) Relaxed definition: cysteine-rich ≥4 cysteines**				
	**Species**	**C-rich total (%)**	**SSPs (%)**	**SSCPs (%)**
Overall	36	60	47	21
Secretome size				
class 1	14	61	41	16
class 2	16	61	49	23
class 3	6	57	57	26
Phylogeny				
Oomycota	3	56	56	21
Saccharomycotina	9	60	42	15
Pezizomycotina	11	65	47	24
Basidiomycota	8	59	48	22
Lifestyle				
saprophyte	15	62	43	20
plant pathogen	11	58	52	23
animal pathogen	8	62	44	18

Numbers of species and average contents of total cysteine-rich proteins, SSPs and SSCPs in total secretomes in secretome size-, phylogenetic and lifestyle-groupings containing at least three species; SSP & SSCP ≤ 300 residues without signal peptides.

### Identification of protein clusters by Markov chain clustering

**Figure 4:**
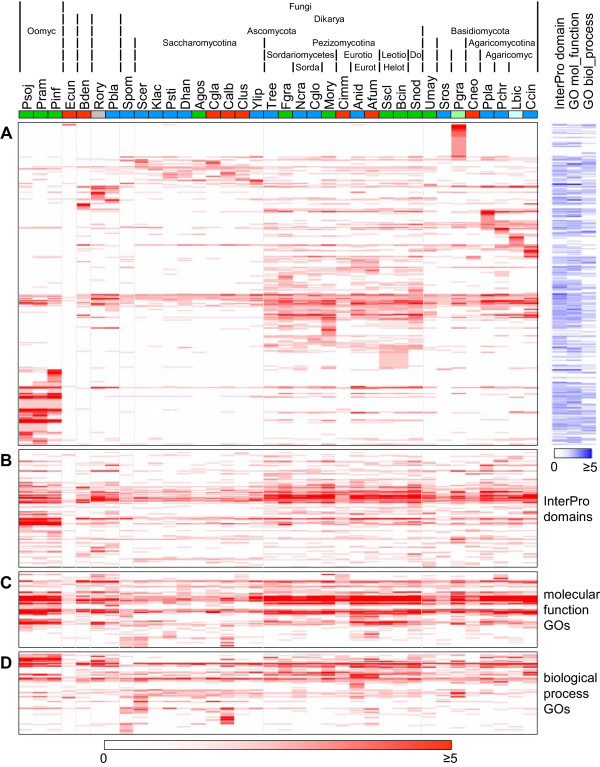
**TRIBE-MCL-clustering of proteins and distributions of InterPro domains and molecular function and biological process GO terms.** Heatmap of protein clusters with at least two proteins **(A)** and InterPro domains **(B)**, molecular function GO terms **(C)** and biological process GO terms **(D)** with at least two occurrences by protein clusters/InterPro domains/GO terms (rows) and species (columns). Increasing number of proteins/occurrences per cluster/InterPro domain/GO term and species is indicated by higher intensity of red. Species (columns) are color-coded by lifestyle: dark blue = saprophytes, dark green = plant pathogens, red = animal pathogens, grey = “polyphage (*R. oryzae*), light blue = Ectomycorrhiza (EcM, *L. bicolor*), light green = obligate biotroph (*P. graminis*). Species abbreviations are given in Table 1. Protein clusters **(A)** with assigned InterPro domains, molecular function or biological process GO terms are provided on the right where the intensity of the blue color indicates their abundances. The heights of the heatmaps correspond to the numbers of individual protein clusters/InterPro domains/GO terms represented in the respective maps.

All soluble secreted proteins identified were subjected to an “all-versus-all” BlastP and this result was then used by the software TRIBE-MCL to arrange all sequences into protein clusters [28]. Thus, 17,610 out of 24,757 proteins were sorted into 2,414 clusters (Figure 4A, Additional file 3A), leaving 7,147 singletons (Additional file 3B). As similarly seen for whole proteomes [29], certain clusters were either abundant in Pezizomycotina or almost specific to them, as visualized by the intensity of red color, e.g. below the universal clusters in the middle of Figure 4A. Other protein clusters were either enriched in Saccharomycotina (Figure 4A, upper region), or practically absent. Furthermore, we identified a large group of Oomycota-specific clusters (Figure 4A, lower left corner), and a number of clusters specific to individual species.

To assign putative functions, all sequences were analyzed with InterProScan [30]. For the annotation of the secreted proteins, only InterPro domains were considered. On average, 32% of the sequences had at least one InterPro (IPR) domain assigned. The highest percentages of annotated proteins were obtained for *Aspergillus nidulans*, *Aspergillus fumigatus*, *Cryptococcus neoformans* and *Trichoderma reesei* (43% each), whereas *P. graminis*, *B. dendrobatidis* and *Encephalitozoon cuniculi* had the lowest percentages (14%, 20% and 20%, respectively), possibly relating to the intensity of research devoted to different taxa. To assign InterPro domains to the protein clusters determined by TRIBE-MCL, InterPro domains found in members of a given cluster were assigned to the entire cluster if at least 75% of the sequences contained it. Using this approach, 515 out of 2,414 protein clusters (21.3%) had at least one InterPro domain assigned (Figure 4A, blue column labeled “InterPro domain”).

Moreover, we obtained Gene Ontology annotations of the types molecular function (mf GO) and biological process (bp GO) for the secreted proteins from the UniProtKB database. On average, 35% of all sequences had at least one molecular function GO annotation and 21% had at least one biological process GO annotation. For the molecular function GO terms, the most extreme values for ratios of proteins in putative secretomes annotated with at least one term were 68% and 65% in *Candida albicans* and *S. pombe*, respectively, and 8% and 14% in *Postia placenta* and *P. graminis*, respectively, whereas for biological process GO terms, the most extreme values were 79% and 76% of putative secretome proteins in *Saccharomyces cerevisiae* and *S. pombe*, respectively, and 9% in both *P. graminis* and *B. dendrobatidis*. As for the InterPro domains, we assigned GO molecular function and biological process annotations to protein clusters when at least 75% of the proteins in a cluster shared the GO term. Thus, of the total of 2,414 protein clusters, 483 were assigned at least one molecular function GO term and 285 were assigned at least one biological process GO term (Figure 4A, blue columns labeled “GO mol_function” and “GO biol_process”).

We then analyzed whether certain protein clusters determined above by TRIBE-MCL occur in all species of a given phylogenetic lineage but do not occur in any species outside of this lineage (Additional file 4A). No cluster existed, that occurred in all 33 fungi. Two clusters contained proteins from all species analyzed except *E. cuniculi*, which is an intracellular pathogen of mammals exhibiting a strongly reduced genome and hence secretome size (Additional file 1). The proteins grouped in those two clusters were disulphide isomerases (cluster 16; IPR012335, Thioredoxin fold; IPR013766, Thioredoxin domain; IPR006662, Thioredoxin-related; mf GO:0015035, protein disulfide oxidoreductase activity; bp GO:0045454, cell redox homeostasis; bp GO:0006662, glycerol ether metabolic process), involved in protein folding and maturation in the ER [31] and undefined proteins (cluster 37; no InterPro domain; mf GO:0005524, ATP binding; no bp GO term). Interestingly, no cluster existed that was absolutely Ascomycota-specific (Additional file 4A). The most widely represented cluster within Ascomycota contained proteins from 20 of the 21 species analyzed within this taxon but was not specific to it since it also occurred in other phyla. The corresponding protein was a mannosyl oligosaccharide glucosidase (cluster no. 110; no InterPro domain; mf GO:0004573, mannosyl-oligosaccharide glucosidase activity; bp GO:0009311, oligosaccharide metabolic process), the first enzyme in the *N*-linked oligosaccharide processing pathway, which was found exactly once per species except in *Chaetomium globosum*.

For Saccharomycotina, Pezizomycotina, and Basidiomycota, one specific cluster each was found (Additional file 4A). The Saccharomycotina-specific cluster (no. 320) comprised two proteins each in *Pichia stipitis* and *C. albicans* and one protein in each of the other species. The cluster had no InterPro domain or mf GO or bp GO term assigned, but 8 of 11 proteins (73%) contained a CFEM (Common in Fungal Extracellular Membrane proteins) domain according to InterProScan that was described as an extracellular N-terminal domain comprising eight cysteines and that is found in certain fungal cell-surface integral membrane proteins [32]. Manual analysis showed that ten proteins belonging to cluster no. 320 contained an authentic CFEM domain, while one sequence had only six cysteines. Only one of these proteins belonged to the SSCPs conforming to our new stringent definition given above. The CFEM domain is also common in Pezizomycotina and Basidiomycota, but the corresponding proteins distribute to other clusters, none of which shows full coverage of a specific phylogenetic lineage as cluster 320 does. Overall, 79 CFEM domains were identified in proteins from 11 protein clusters and one orphan protein.

The Pezizomycotina-specific cluster no. 240 (no InterPro domain or mf GO or bp GO term) was represented by two proteins in *T. reesei*, *Fusarium graminearum,* and *Neurospora crassa,* and by one protein in each of the other species. According to our above definition, all these proteins were SSCPs. The single Basidiomycota-specific cluster no. 190 (no InterPro domain or mf GO or bp GO term) comprised one to three members in each of the species analyzed.

We analyzed if certain clusters were exclusively associated with all species exhibiting a given nutritional lifestyle (Additional file 4B). We detected one particular cluster (no. 469; no InterPro domain, mf GO or bp GO term) that was found in all plant-pathogenic Pezizomycotina but neither in any other species of that subphylum having a different lifestyle nor in plant pathogens outside of the Pezizomycotina. The saprophytic Agaricomycetes *Coprinus cinereus*, *Phanerochaete chrysosporium,* and *P. placenta* had two clusters that encompassed only these species, i.e. no. 592 (no InterPro term; mf GO:0008236, serine-type peptidase activity; no bp GO term) and no. 710 (no InterPro domain or mf GO or bp GO term).

On average 60% of the sequences in each secretome clustered with sequences from other species and another 11% with other sequences exclusively from the same species. Despite this general trend, extreme examples existed where only very small fractions of the secretomes found similar members in other species. In particular, only 20%, 9% and 14% of the sequences in the secretomes of *B. dendrobatidis*, *E. cuniculi,* and *P. graminis* did so, while 59%, 21%, and 37% clustered exclusively within these species, respectively. This suggested that large parts of their secretomes are specific for these organisms or the lineages they belong to. Importantly, the heatmap representation of the TRIBE-MCL results indicated that proteins rather cluster by phylogeny than by nutritional lifestyle.

### Analysis of occurrence and abundance of InterPro domains and molecular function and biological process GO terms

Above, we assigned InterPro domains and molecular function and biological process GO terms identified in the proteins of a cluster to that cluster only if these were identified in at least 75% of the proteins forming that cluster. To analyze the secretomes based on functional traits, irrespective of the clustering of the proteins carrying them, we evaluated the presence and abundance of the InterPro domains and GO terms in relation to phylogeny and lifestyle. From totals of 12,716, 13,355 and 6,556 occurrences of InterPro domains, molecular function and biological process GO terms, respectively, around 97% of each were derived from multiple occurrences of 548, 352 and 331 individual terms. For comparison, only 17,610 of 24,757 proteins (71%) were members of any of 2,414 clusters. Moreover, the 17,610 proteins in clusters accounted for 90% each of all occurrences of the three annotation types (Table 3). In the heatmap representations of abundances of InterPro domains, molecular function and biological process GO terms (Figures 4B, C and D), distribution patterns were similar to protein distribution patterns (Figure 4A) but they were less prominent. For example, the band that spreads across the entire species spectrum in the middle of the protein cluster heatmap corresponds to the region roughly one third from the top in the InterPro domain heatmap. This band appears broader for the InterPro domains (Figure 4B) than for the protein clusters (Figure 4A), because many of the highly conserved proteins in these clusters contain several different, equally conserved InterPro domains. The Saccharomycotina-enriched protein clusters in the upper region of the protein cluster heatmap (Figure 4A) are mirrored by Saccharomycotina-enriched InterPro domains in the lower region of the respective heatmap (Figure 4B). Similarly, species-specific protein clusters for *B. dendrobatidis*, *R. oryzae*, *Phycomyces blakesleeanus*, *P. graminis,* and *P. placenta* in the upper region of Figure 4A correspond to specific InterPro domains for these species in the lower and upper regions of Figure 4B. Similar comparisons can be made for the molecular function and biological process GO terms as well (Figures 4C and D). Interestingly, a few species show many more GO annotations than all others do, e.g. *S. pombe*, *S. cerevisiae* and *C. albicans*, caused both by high ratios of the predicted secretomes having at least one annotation and many individual proteins having many different annotations.

**Table 3:** **Distribution of proteins, InterPro domains and GO terms**

	Proteins	InterPro domains	Molecular function GOs	Biological process GOs
Total	24,757	12,726	13,355	6,556
In any protein cluster	71%	89%	90%	90%
In protein clusters ≥3 species	53%	82%	83%	83%
Terms occurring ≥ twice		97%	98%	96%
Terms occurring in ≥3 species		95%	97%	87%

Total numbers of proteins and of InterPro domain, molecular function GO and biological process GO term occurrences identified in these proteins. Fractions of proteins contained in protein clusters with at least two proteins and in clusters with proteins from at least three species. Fractions of InterPro domain, molecular function GO and biological process GO term occurrences identified in these protein clusters and fractions of total occurrences of these terms derived from individual terms occurring at least twice and in at least three species.

Next, we analyzed the InterPro domains, i.e. functional features, for correlation with phylogeny or nutritional lifestyle. We identified no InterPro domain present in all 36 species, but five existed in all species except *E. cuniculi* (Additional file 4C). Four of these five relate to disulfide-dithiol-mediated redox regulation. Most individual occurrences of the four were identified in the proteins making up the disulphide isomerase protein cluster 16 (IPR012335, Thioredoxin; IPR013766, Thioredoxin domain; IPR006662, Thioredoxin-related; mf GO:0015035, protein disulfide oxidoreductase activity; bp GO:0045454, cell redox homeostasis; bp GO:0006662, glycerol ether metabolic process; see Additional file 4A). The fifth InterPro domain present in all species except *E. cuniculi* represented a domain of glycoside hydrolases (IPR013781, Glycoside hydrolase, catalytic core). No other InterPro domains were fully conserved within wide phylogenetic lineages (Additional file 4C). The two Zygomycota exclusively shared two InterPro domains, whereas the three Oomycota shared 12. Comparing InterPro domains to lifestyle, no correlation was observed at all (Additional file 4D). Three molecular function GO terms were conserved among all 36 species (GO:0046872, metal ion binding; GO:0005524, ATP binding and GO:0008270, zinc ion binding), while by phylogeny, five others were conserved in all species except *E. cuniculi* (GO:0004553, hydrolase activity, hydrolyzing O-glycosyl compounds; GO:0004252, serine-type endopeptidase activity; GO:0030246, carbohydrate binding; GO:0005509, calcium ion binding; GO:0015035, protein disulfide oxidoreductase activity; Additional file 4E).

There existed only a single biological process GO term conserved in all species (GO:0006457, protein folding), while two others (GO:0045454, cell redox homeostasis and GO:0006662, glycerol ether metabolic process) were conserved in all species except *E. cuniculi* (Additional file 4G). By lifestyle, no molecular function or biological process GO term was conserved (Additional files 4 F and H).

### Protein cluster contractions and expansions

**Figure 5:**
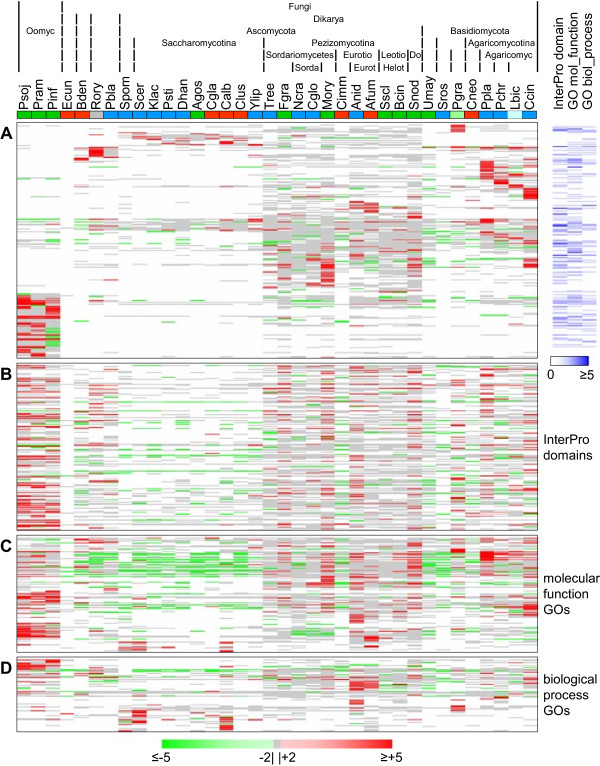
**Expansions and contractions in protein clusters and in InterPro domain and molecular function and biological process GO term abundances.** Heatmap of protein clusters **(A)**, InterPro domains **(B)**, molecular function GO terms **(C)** and biological process GO terms **(D)** represented in at least three species that show an expansion of more than twofold (red) or a contraction of less than half (green) of the median of the abundances per species for the respective entry type. Increasing level of expansion or contraction per cluster/InterPro domain/GO term and species is indicated by higher color intensity. Less than twofold expansion or contraction is shown in monochrome grey. White indicates no protein in this cluster or occurrence of this InterPro domain or GO term in this species. Species (columns) are color-coded by lifestyle: dark blue = saprophytes, dark green = plant pathogens, red = animal pathogens, grey = polyphage (*R. oryzae*), light blue = Ectomycorrhiza (EcM, *L. bicolor*), light green = obligate biotroph (*P. graminis*). Species abbreviations are given in Table 1. Protein clusters **(A)** with assigned InterPro domains, molecular function or biological process GO terms are provided on the right where the intensity of the blue color indicates their abundances. The heights of the heatmaps correspond to the numbers of individual protein clusters/InterPro domains/GO terms represented in the respective maps.

So far, we analyzed the absence or presence of protein clusters in all fungi sharing a given phylogenetic lineage or lifestyle. However, protein families may have contracted or expanded in individual species or parts of evolutionary lineages and such changes may relate to lifestyle. To reduce noise in quantitative assessment, expansion was defined as the presence of more than twice the median number of proteins for a cluster in a given species, contraction as less than half the median number of proteins. Applying these criteria to the 902 protein clusters present in at least three species, that thus contain at least three proteins to be able to calculate medians, we found 251 clusters with expansions and/or contractions in at least one species (Figure 5A; expansions are coded red and contractions green). Within the limits of the applied criteria, no protein cluster was contracted or expanded exclusively in all species belonging to a certain phylogenetic lineage, or exhibiting a certain nutritional lifestyle (Additional file 5A, B). Even when scoring contractions or expansions less stringently, i.e. when allowing that this might have happened in additional species representing other lineages or lifestyles, we only found one protein cluster. This cluster (no. 15; no InterPro domain or GO term) was expanded in all five Agaricomycotina, being present in five copies in the mammalian pathogen *C. neoformans* and up to seventeen in the saprophyte *P. placenta*. In other Basidiomycota, it was expanded in the cereal pathogen *P. graminis* (ten copies), in the maize pathogen *U. maydis* (eight copies), but not in the saprophyte *S. roseus* (one copy). This is visualized in Figure 5A by a red line just below the empty upper region in the Basidiomycota that is gapped only under *S. roseus*. Beyond, cluster 15 was also expanded in the Zygomycota *P. blakesleeanus* (saprophyte) and *R. oryzae* (polyphage), which had six and sixteen proteins, respectively. It had just one copy in four species of Saccharomycotina whereas the remaining species entirely lacked it. Except in *C. globosum* where it was also missing, species in the Pezizomycotina had up to three proteins belonging to cluster 15.

The lack of general contractions even when using the less stringent condition results probably from the fact that in most clusters the median of the number of proteins was very low (between 1.0 and 1.8) and that the absence of a protein in a species was not scored as a contraction. However, we did observe in five instances interesting trends of expansions or contractions in certain phylogenetic lineages (Additional file 6). The first of these five protein clusters (no. 15; no InterPro domain or GO term) was described above as the only one showing a general expansion in a phylogenetic lineage (Agaricomycotina) and some Basidiomycota and Zygomycota.

The second (cluster no. 65; no InterPro domain or GO term), apparently arose in the Ascomycota since it was not present in any of the other lineages. The Saccharomycotina had between two and five members in each species, whereas in the Pezizomycotina the protein number was contracted to one each in *F. graminearum*, *M. oryzae*, *Coccidioides immitis*, *A. nidulans*, and *A. fumigatus*, visible as a green line in the uppermost part of Figure 5A. *T. reesei*, *C. globosum*, *S. sclerotiorum*, and *S. nodorum* lacked these proteins completely.

The third protein cluster (no. 48; no InterPro domain or GO term) showed a trend of contraction in Agaricomycetes. Within Basidiomycota, members of this cluster were restricted to species of this class with only one copy each, except *P. placenta* that had five. In the Pezizomycotina, it was found with two to six proteins per species, except in *C. immitis* that had only one. All other lineages entirely lacked cluster 48.

The fourth cluster (no. 64; no InterPro domain or GO term) showed trends of expansion in three Pezizomycotina. It contained proteins in each Basidiomycota except *U. maydis* and in each Pezizomycotina except *F. graminearum*, *M. oryzae,* and *C. immitis*. The Basidiomycota and *T. reesei*, *N. crassa*, *C. globosum*, *S. sclerotiorum* and *S. nodorum* each had one to four of such proteins. The Helotiales *B. cinerea*, the Eurotiales *A. nidulans* and *A. fumigatus* showed expansions with five, five, and seven copies, respectively. All other lineages entirely lacked cluster 64 (Additional file 6).

Finally, cluster 134 (no InterPro domain or GO term), was also only found in Pezizomycotina and Basidiomycota. In Basidiomycota, only *U. maydis* and *C. cinereus* had this cluster, containing one and three members, and in Ascomycota only seven species within the Pezizomycotina with up to six copies in *S. nodorum*. The Eurotiales *A. nidulans* and *A. fumigatus*, but also *U. maydis* showed a contraction in cluster 134, with one copy each.

When further focusing our analysis on individual species, we observed a number of expanded protein clusters on condition that a given cluster should comprise at least ten members in that species and at least 50% more members than in all other species having this cluster as well (Additional file 7). Disregarding the *Phytophthora* species, 21 of such cases were found in species of Basidiomycota and Saccharomycotina, but not of Pezizomycotina, and further in *B. dendrobatidis* and *R. oryzae*. In general, members of Basidiomycota showed the most extreme cases of such single-species expansions (Additional file 7).

### Contractions and expansions in abundance of InterPro domains and of molecular function and biological process GO terms

As we analyzed the presence or absence of all InterPro domains and molecular function and biological process GO terms identified in the 36 secretomes independent of the proteins in specific lineages or lifestyles, we also analyzed whether specific contractions or expansions occurred in these data sets. We applied the same limits for contraction or expansion of abundances of these terms as for protein clusters. Thus, out of 412 InterPro domains, representing a total of 12,035 individual InterPro domain occurrences, 177 showed a contraction or expansion in at least one species (Figure 5B). Furthermore, out of 256 and 177 molecular function and biological process GO terms, representing 13,150 and 5,722 individual occurrences, 124 and 78 showed at least one expansion or contraction, respectively (Figures 5C and D). As for protein clusters, abundance of no InterPro domain was contracted or expanded exclusively in all species from any phylogenetic lineage or specific nutritional lifestyle (Additional file 5C, D) or subgroup thereof (for example necrotrophic vs. non-necrotrophic plant pathogens). Nevertheless, we observed trends of contraction or expansion. These, however, were mainly identified in the context of phylogeny. For those InterPro domains that were identified exclusively (18 cases) or to at least 75% (25 cases) in the members of individual protein clusters, expansions and/or contractions of InterPro domain abundances in multiple species (10 and 18 cases, respectively) typically paralleled the expansions and/or contractions in the corresponding protein clusters (Additional files 8B and C, respectively). The remaining, however, did not show such one-to-one associations (Additional file 8A) and in the following, we highlight some noticeable cases.

IPR013781 (Glycoside hydrolase = GH, catalytic core) was the most common domain in the data set and is linked to a large number of GH families. This domain was identified in all species except *E. cuniculi* and within Fungi was mostly far more abundant in Pezizomycotina, with 5 to 44 occurrences per species, and Basidiomycota, with 5 to 38 occurrences per species, than in Saccharomycotina, where it was contracted with three to nine occurrences per species. Interestingly, *B. dendrobatidis* contained IPR013781 only once. The animal pathogens *C. immitis* (Pezizomycotina) and *C. neoformans* (Basidiomycota) and the saprotrophic Basidiomycota *S. roseus*, with five or eight occurrences, also showed contractions for this InterPro term within their phylogenetic lineages. Of all the other InterPro domains linked to IPR013781, only IPR001223 (Glycoside hydrolase, family 18, catalytic domain) and IPR001547 (Glycoside hydrolase, family 5) showed similar distributions (Additional file 8A). The latter is contracted in Saccharomycotina. Not surprisingly, molecular function GO terms correlating with IPR013781, particularly mf GO:0004553, hydrolase activity, hydrolyzing O-glycosyl compounds, the most common molecular function GO term in the data set, and mf GO:0016787, hydrolase activity, showed similar patterns of contractions. These terms, however, also showed expansions in many Pezizomycotina and Basidiomycota, but no correlation with lifestyle existed for these expansions.

As glycoside hydrolases are enzymes important for such diverse aspects as cell wall remodeling, degradation of host cell walls by plant pathogens to allow spreading through tissues and degradation of polysaccharides to provide carbon sources, all secreted proteins were analyzed against the CAZy database by dbCAN. In total, members of 71 GH families were identified (Additional file 9). Whereas 61 and 45 GH families were identified in Pezizomycotina and Basidiomycota, respectively, Saccharomycotina contained members in only 21 GH families. In Pezizomycotina, GHs ranged from 25 proteins in 16 families in the mammalian pathogen *C. immitis* to 119 proteins in 42 families in the plant pathogen *S. nodorum*. In Saccharomycotina, GH content ranged from 14 proteins in 10 families in the mammalian pathogen *Candida lusitaniae* to 28 proteins in 15 families in the mammalian pathogen *C. albicans*. In Basidiomycota, GHs ranged from 14 proteins in nine families in *S. roseus* to 90 proteins in 31 families in *C. cinereus*. With respect to nutritional lifestyle, GH content was relatively low in the specialized animal pathogens *C. neoformans* (26 proteins in 13 families) and *C. immitis* (25 proteins in 16 families), but also in the biotrophic plant pathogen *U. maydis* (40 proteins in 25 families) and in the saprophytic yeast *S. roseus* (14 proteins in 9 families).

IPR012334 (Pectin lyase fold), the second most abundant InterPro domain in the entire data set (Additional file 8A), is a domain that is found in pectate lyase, iota-carrageenase and GHs from family 28, including various galacturonases. Within Dikarya, it was absent from Saccharomycotina except for contracted single occurrences in *S. cerevisiae* and *Ashbya gossypii*, from *S. pombe*, and from *S. roseus*. In Pezizomycotina, it was typically more abundant than in Basidiomycota. Expansion was seen in Eurotiales, in Helotiales and in *F. graminearum*.

IPR000834 (Peptidase M14, carboxypeptidase A) was present in all Ascomycota except *Debaryomyces hansenii*, but only in four of eight Basidiomycota (*U. maydis*, *S. roseus*, *L. bicolor* and *C. cinereus*). Within Pezizomycotina, it was expanded in all plant pathogens. This domain is contained in zinc-dependent metalloproteases from family M14 that are typically soluble secreted proteins, synthesized as zymogenic proproteins, that degrade proteins by hydrolyzing single residues from the C-termini (MEROPS; http://merops.sanger.ac.uk/cgi-bin/famsum?family=M14).

IPR001002 (Chitin-binding, type 1) describes an N-acetylglucosamine-binding domain that occurs in chitinases of fungi and plants. It is ubiquitous in Pezizomycotina except *C. immitis* that lacks it, and is expanded in all plant pathogens. In both Saccharomycotina and Basidiomycota, however, only half of the species share it and in each, it is contracted with one occurrence only. As expected, the corresponding molecular function GO term, GO:0008061, chitin binding, shows practically the same pattern of abundances, contractions and expansions. Interestingly, though, the expansions of chitin-binding domains in a number of mostly plant pathogenic Pezizomycotina was not mirrored by the terms mf GO:0004568, chitinase activity and bp GO:0006032, chitin catabolic process, relating to enzymatic activity. Either these species may have some chitin-active enzymes containing multiple chitin-binding domains, or these domains may occur independently of enzymes, for example in effectors shielding the fungi from recognition by their host plants.

The last example of expansions in abundance of InterPro domains are IPR000172, GMC oxidoreductase, N-terminal and IPR007867, GMC oxidoreductase, C-terminal. Glucose methanol choline oxidoreductases are flavoenzymes utilizing various substrates. Within Fungi, these InterPro domains were only found in Pezizomycotina and Basidiomycota. Interestingly, while these domains tend towards expansion in the plant pathogenic Pezizomycotina they are lacking in the specialized animal pathogens *C. immitis* and *C. neoformans*, respectively. *C. cinereus* has at least thrice as many occurrences of both GMC oxidoreductase InterPro domains as any other Basidiomycota species.

As for protein clusters, we also observed a number of InterPro domains, molecular function and biological process GO terms that showed major expansions specifically in a single species, with more than ten occurrences and at least 50% more occurrences than in any other species having that InterPro domain. Many of these expansions reflected expansions in the protein clusters, annotated with these InterPro domains or GO terms (Additional file 10). However, some prominent examples existed of expanded terms not linked to expanded protein clusters, such as IPR015880 (zinc finger, C2H2-like) in the Pezizomycotina *M. oryzae* with 12 occurrences, and IPR000772 (ricin B lectin) in the Basidiomycota *C. cinereus* with 13 occurrences.

## Discussion

The completion of annotated proteomes allows for in depth analyses of secretomes by bioinformatics. Our comparative analyses of fungal and Oomycota secretomes, representing a broad range of taxa and nutritional lifestyles, revealed that primarily phylogenetic relationships determine the size and the composition of the secretomes, and to a lesser extent the nutritional lifestyle. We identified protein clusters that are specific for or expanded in certain lineages. Others are expanded with respect to lifestyle, but only within individual lineages.

### Secretome size and composition

Secretome size correlates with phylogenetic lineages. Saccharomycotina have smaller secretomes, both absolute and relative, than Pezizomycotina or Basidiomycota, which have similar sized secretomes in both respects. This may be because yeasts are less capable of utilizing complex organic polymers as substrates, since they often live in niches providing access to sugars and other compounds that are easier to metabolize.

Secretome size is also associated with lifestyle. Plant pathogens have the largest, saprophytes have intermediate, and animal pathogens have the smallest secretomes. To some extent, this finding may be explained by the assumption that animal hosts represent a nutritionally simpler environment than plant hosts or litter. Furthermore, plant pathogens, particularly obligate biotrophs such as *P. graminis*, may secrete many effectors that would not provide benefits to saprophytes. It was suggested that portions of their secretomes participate in the coevolutionary arms race with host factors thus leading to sequence diversification [33]. Besides, *P. graminis* initially infects its alternate host *Berberis* to switch thereafter to a cereal host. It must be presumed that the sets of secreted proteins involved in infection of both hosts do only partially overlap which will further increase the total size of the rusts’ secretome. Moreover, the typically small size of animal pathogen secretomes may reflect an adaptation to the flexibility of the animal immune system. Against a system effectively recognizing non-self molecules and mounting system-wide defense reactions in a circulatory system, a “stealth mode” is likely an efficient way of evasion. In such a scenario, it appears as a selective advantage to minimize the numbers of putative PAMPs and thus to reduce the size of the secretome.

Comparison of the mammalian pathogens within Ascomycota points to another link between environmental influences and secretome size. The three *Candida* species, *C. immitis* and *A. fumigatus* have secretomes of 203-286, 384 and 676 proteins, respectively. Besides causing invasive mycoses in immunocompromised hosts, *Candida* species are commensals on mammalian skin and mucosa. *C. immitis* is able to infect immunocompetent hosts and beyond lives as a saprophyte on host carcasses, with a concomitant lack of many enzymes required for decomposition of plant materials [34]. *Aspergillus* species typically are environmental saprophytes and only a few of them can in addition act as opportunistic pathogens of humans and animals [35]. Comparison of the two *Aspergillus* species in our data sets shows that *A. nidulans*, having only the saprophytic lifestyle, has absolutely and relatively a larger secretome, and secretes 145 more enzymes than *A. fumigatus*. These examples provide support for the notion that dominance of the saprophytic phase is associated with a larger secretome. Furthermore, the higher number of secreted enzymes allows *A. nidulans* to utilize a wider variety of substrates present in soil or plant debris, which are likely more difficult to degrade than those available in animal hosts. In addition, competition with other microorganisms is conceivably less severe in animal hosts than in soil or litter, which may affect the number of secreted proteins involved in combatting competitors.

The secretomes in the Oomycota are considerably larger than in most Pezizomycotina and Basidiomycota and larger than most fungal plant pathogen secretomes in absolute, but not in relative numbers. The high absolute numbers of secreted proteins are derived, at least in part, from expansions in the RXLR- and Crinkler-effector families which comprise a considerable fraction of these secretomes [36].

We analyzed the secretomes for the distribution of size classes and cysteine richness to determine whether SSCPs, previously discussed to harbor many effectors, are enriched in certain phylogenetic lineages or nutritional lifestyles. Examples for this type of effectors are some well characterized proteins from the biotrophic plant pathogen *Cladosporium fulvum* [37]. Other SSCPs are hydrophobins covering aerial mycelia and spores [38], the membrane-bound CFEM-proteins [32], antimicrobial proteins important to fight off competitors [39] and peptides such as PtrToxB of *Pyrenophora tritici-repentis* targeting the host [40]. Using our definition of SSCPs (≤300 residues mature, ≥4 Cys in total and ≥5% Cys overall) our analysis shows a correlation of both phylogeny and lifestyle with the percentage of SSCPs in secretomes. By phylogeny, the relative SSCP content is similar in Pezizomycotina and in Basidiomycota, while it is almost four-fold lower in Saccharomycotina. As for lifestyle, the SSCP fraction of the total secretomes is 30% higher in plant pathogens than in saprophytes, presumably because of the considerable numbers of potential effectors. In animal pathogens, this fraction is only half of that of saprophytes. The fact that the highest individual SSP and SSCP fractions of the total secretomes occur in *P. graminis* and the hemibiotrophic Pezizomycotina *M. oryzae* fits with the assumption that effectors would play eminent roles particularly in biotrophy. Our results correspond well with the large secretomes and high numbers of SSPs recently reported for obligate biotrophic phytopathogens *Melampsora larici-populina* and *P. graminis* [25, 41].

### Presence/absence of and contractions/expansions in protein clusters, InterPro domains and GO terms

We clustered all proteins into families, assigned InterPro domain and molecular function and biological process GO annotations, and identified clusters with expansions or contractions in specific taxonomic groups. It is important to keep in mind that what we define here as an expansion may be a recent lineage specific series of gene duplications or could be due to ancient gene duplications followed by gene loss in the other lineages. To distinguish between these two alternative hypotheses will require in depth phylogenetic analyses of each family. However, in either case, we consider that an expansion represents the presence of lineage specific selective pressure for those species to maintain the gene, regardless of whether the gene duplication events were recent or ancient. The secretomes of the Saccharomycotina lack many protein clusters completely, which may relate to their unicellular lifestyle and overall reduced genomes. Some of the clusters missing in Saccharomycotina are expanded or contracted in Basidiomycota compared to Pezizomycotina or *vice versa*. Apart from reflecting the specific phylogenetic histories of the corresponding species, expansion of protein families may correlate with their abilities to cope with more complex and/or variable environments.

Assessment of distinct phylogenetic lineages revealed that Saccharomycotina, Pezizomycotina, and Basidiomycota each contain a single lineage-specific protein cluster. The Saccharomycotina-specific cluster contains the CFEM motif, but narrowly misses the threshold for InterPro domain assignment and has no GO terms assigned. The CFEM domain is not Saccharomycotina-specific, as we identified it in 12 protein clusters across the Dikarya. This example illustrates the intrinsic difficulties in analysis of protein functions by assigning InterPro domains or GO terms to protein clusters. Some clusters have not been assigned terms because the assignment threshold was missed or proteins containing the same terms were clustered in different protein clusters. For this reason, we also performed analyses for the InterPro domains and molecular function and biological process GO terms themselves. Four of five InterPro domains absent only in *E. cuniculi* relate to ER- or Golgi-localized proteins, which may reflect the major reduction in secretion in this intracellular parasite [42]. The fifth represents the catalytic core of glycoside hydrolases. No other InterPro domain showed absolute specificities either by phylogeny or by lifestyle.

By our definition, we found no protein cluster or InterPro domain either contracted or expanded exclusively within all species representing a particular lifestyle or phylogenetic lineage. Nevertheless, five protein clusters were expanded or contracted in some species in certain phylogenetic lineages. For InterPro domain abundances, we found two terms to be contracted in Saccharomycotina and further observed trends of expansions, as well as contractions, in subsets of species within phylogenetic lineages. In Saccharomycotina, the Glycoside hydrolase catalytic domain (IPR013781) and Glycoside hydrolase, family 5 (IPR001547) were contracted according to the definition applied, consistent with the GO- and particularly the CAZy-data. This may be indicative of a narrower spectrum of exploitable polysaccharides in this taxon.

Two domains are expanded in all plant pathogenic Pezizomycotina. Some of the zinc-metalloproteases from family M14 (IPR000834), that typically degrade extracellular matrix proteins, may be involved in degradation of plant tissue, others however could mediate the suppression of plant defense by degradation of host proteins involved in pathogen recognition in the apoplast or playing other important roles in defense. The other expanded IPR domain is the chitin-binding domain, type 1 (IPR001002) that occurs in single or multiple copies in fungal chitinases and other glycoside hydrolases. In plant pathogenic fungi, it may mediate binding of the containing proteins to the fungal cell wall to avoid host recognition by shielding or modifying chitin, which is a PAMP as discussed above. In addition, chitinases or other glycoside hydrolases containing this domain could serve host cell wall degradation or fungal morphogenesis.

Several InterPro domains for glycoside hydrolases and other enzymes involved in degradation, as well as GO terms relating to such activities, show considerable tendencies towards expansion in saprophytes and plant pathogens in the Pezizomycotina and Basidiomycota. For glycoside hydrolases, the CAZy-data confirmed this tendency. The likely explanation is that these species can utilize a wider variety of complex carbohydrates than yeasts or animal pathogens. Examples are IPRs 000172 and 007867 (GMC oxidoreductase domains) that are particularly expanded in plant pathogenic Pezizomycotina plus the saprotrophic Basidiomycota *C. cinereus*. Proteins containing these domains, including various alcohol and sugar peroxidases, produce hydrogen peroxide, which may amongst others modify target molecules directly or serve as substrate for oxygen radical generation by Fenton chemistry. Radicals may serve the breakdown of lignin in white rot fungi or of polysaccharides as in cellulose degradation by brown rot fungi [43]. The role of peroxide generated by plant pathogens is diverse [44]. For example, it may interfere with plant defense compounds by direct reduction, as was recently proposed for a FAD-dependent glucose dehydrogenase from the Pezizomycotina *Glomerella cingulata* that causes anthracnose on various fruits [45].

Apparently, only few protein clusters specific for lineages exist, which may be explained by the broad phylogenetic distance covered by the fungi analyzed thus obstructing the clustering of related proteins. In addition, particularly in the case of plant pathogenic fungi, proteins required for host interaction may be under positive selection due to the “arms-race” between host and pathogen [46]. Thus they may not be recognized as originating from the same ancestral gene anymore, even between relatively closely related species as shown for three species of powdery mildew fungi that only shared less than 10 effectors [47]. Finally, losses or gains of protein families need not be equivalent with losses or gains of biological functions, as these may be provided by other protein families. It has been shown that changes in lifestyle (saprotroph-to-pathogen and reverse) have occurred often in fungal evolution [48–51] and such events need not be accompanied by the same losses or gains of proteins.

With respect to expansion of protein clusters in individual species only, we observed for example an expansion of peptidases (zinc-dependent metallopeptidases of family M36 that hydrolyze matrix proteins (http://merops.sanger.ac.uk/cgi-bin/famsum?family=M36) and serine endopeptidases of family S41 that cleave target proteins close to alanine-rich C-terminal tripeptides (http://merops.sanger.ac.uk/cgi-bin/famsum?family=S41)) in *B. dendrobatidis*, confirming previous observations [52]. This may reflect an adaptation to the animal pathogenic lifestyle and similar expansions were observed for peptidases in other fungi exhibiting this lifestyle. A study comparing closely related animal pathogens and saprophytes showed that in *C. immitis* and *C. posadasii* families of subtilisins and other family S8 serine peptidases, which typically are non-specific endopeptidases (http://merops.sanger.ac.uk/cgi-bin/famsum?family=S8), and mentioned M36 metalloproteases are expanded [34]. These families are also expanded in the non-pathogen *Uncinocarpus reesii*, but not in the more distantly related *Histoplasma capsulatum*. In contrast to these Onygenales, within the Eurotiales these peptidase families are expanded neither in the saprophyte *A. nidulans* nor in the opportunistic pathogens *A. fumigatus* and *A. terreus*, illustrating different extents of adaptation to the pathogenic lifestyle. Complementary, whereas the Onygenales show strong contractions in, or even lack of a number of plant cell wall degrading enzyme families (cutinase, cellulose and pectin esterase, among others), the Helotiales all contain similar numbers of these enzymes [34]. This suggests that there is a considerable phylogenetic signal in the data of species comparisons irrespective of the degree of kinships and furthermore, that lifestyles are not linked to the presence, absence or abundance of certain protein families of functional groups in a straight-forward causal relationship.

## Conclusion

We identified and analyzed the soluble secretomes of fungi to test the hypothesis that the secretomes of species belonging to different phylogenetic lineages but sharing the same nutritional lifestyle also share a similar composition. Secretome size correlates with lifestyle, with plant pathogens having the largest and animal pathogens having the smallest secretomes. In contrast, the signal of lifestyle on the specific composition of secretomes is restricted, for which we propose two explanations. First, many of the species analyzed, except the obligate biotrophs, do not follow only one lifestyle throughout their life cycle. For example, even a sophisticated plant pathogen such as *F. graminearum* has an extended saprophytic growth phase during which it has to compete with soil microorganisms that will not play a role during the pathogenic phase. Thus, the secretome of such a fungus will show the imprint of adaptation to several habitats. Second, expansions and contractions of gene families and functional domains reflect a strong phylogenetic signal that may overrule that of lifestyle. However, within each lifestyle, we find expansions in gene families with important roles. In summary, we found only limited evidence for converging evolution to shape the overall composition of fungal secretomes.

## Methods

### Secretome prediction

We retrieved 36 predicted proteomes from the Broad Institute, Joint Genome Institute, Génolevures and NCBI (Additional file 1) and reformatted them as multi-FASTA files, if necessary. Analysis by SignalP2 and 3, and TargetP was performed at http://www.cbs.dtu.dk. Since it had been demonstrated that the accuracy of prediction increased when combining these algorithms [22], we considered proteins to have an authentic signal sequence if the results of the analyses with TargetP, SignalP 2.0 maximum Y-score and SignalP 3.0 maximum S-score were all positive. Analysis by Phobius was performed at http://phobius.sbc.su.se on complete predicted proteomes. All of these analyses applied default parameters. Proteins were defined as soluble secreted when no transmembrane helices were predicted by Phobius. Analyses for protein content in secretomes and for cysteine content were performed in Microsoft Excel. As a cautionary note: the annotated proteome sequences that form the basis of our analyses had originally been predicted by various techniques and may thus be of varying quality and completeness.

### Functional annotation

To identify known motifs and domains, the set of soluble secretome sequences was analyzed using InterProScan (http://www.ebi.ac.uk; database release 17.0) using the stand-alone InterProScan package [53]. InterPro is a database that integrates families, domains, regions and sites from various member databases, into a complex structure with various levels of interdependence (parent/child, contains/found in and overlaps with other InterPro terms independent of these two InterPro interconnection types) [30]. For functional annotation, only InterPro terms of the entry type “domain” were used and protein clusters were assigned IPR domains, when ≥ 75% of the cluster member proteins had obtained the domain. Furthermore, Gene Ontology annotations (GO terms) for the secreted proteins were retrieved from the UniProt knowledgebase (UniProtKB; http://www.uniprot.org) [54]. For annotation, only GO terms of types “molecular function” and “biological process” were used. As for InterPro domains, protein clusters were assigned GO terms, when at least 75% of the cluster member proteins were annotated with that GO term.

The secretomes were analyzed for the presence of carbohydrate-active enzymes by batch blast against the CAZy database (http://www.cazy.org) at dbCAN (http://csbl.bmb.uga.edu/dbCAN/) [55]. Sequences with homologies to glycoside hydrolase (GH) hidden Markov models were considered glycoside hydrolases when the aligned regions covered at least 70% of the hidden Markov models.

### Clustering

The sequences from the complete set of predicted secretomes were compared to each other with an all-vs.-all BlastP search using default parameters [56]. The resulting matrix was used for clustering the sequences with TRIBE-MCL with an inflation parameter value of 1.75 [28]. This somewhat low value was used to account for the facts that phylogenetically, the range of taxa analyzed was very wide and contained, compared to the entire proteomes, fewer highly conserved proteins, which would be clustered even by a higher inflation parameter value. This approach allowed shifting the balance somewhat towards the benefit of an increased sensitivity by easing somewhat on the specificity of analysis as assessed in preliminary test runs. The clustering results were visualized as heatmaps using Java Treeview [57]. The InterPro domains identified by InterProScan [53] in the secretomes as well as the molecular function and biological process GO terms obtained from UniProtKB were ordered with Gene Cluster 3.0 [58]. First, the terms and species were organized with the SOM algorithm using uncentered correlation, average linkage distance metric. Next, the trees were constructed using hierarchical clustering using the same distance metric.

### Analysis of contractions and expansions

Contraction was defined as less than half the number of proteins or InterPro term occurrences compared to the respective cluster or InterPro term occurrence median, expansion as more than twice the median number of proteins or InterPro term occurrences. Analyses were performed in Microsoft Excel and visualized as heatmaps using Java Treeview [57].

## Electronic supplementary material

Additional file 1: **Species data.** Data sources and information on the taxonomy, genome, proteome, and secretome for 33 fungal and three Oomycota species used in this study. (XLS 44 KB)

Additional file 2: **Protein length and cysteine-richness data for soluble secretomes.** Cysteine-rich proteins in the total soluble secretomes by two definitions: (A) stringent (≥4 Cys and ≥5% Cys) and (B) relaxed (≥4 Cys). The absolute numbers and the ratios of all proteins and of small proteins (≤300 amino acids in the predicted mature chains) in the total secretomes and the absolute numbers and the ratios of cys-rich proteins in the two protein length ranges are shown. For Cys-rich small proteins, the ratios within the small proteins are shown as well as the ratios in relation to the total secretomes. Also shown are the overall mean ratios for each group, the mean ratios in the soluble secretome size classes 1 to 3, and the mean ratios by lifestyles saprophytes, plant pathogens, and animal pathogens. Species are ordered by phylogeny. (XLS 88 KB)

Additional file 3: **TRIBE-MCL protein clusters with constituent proteins and orphan proteins.** (A) Protein clusters generated by TRIBE-MCL with numbers of proteins and constituent proteins per cluster. (B) Orphan proteins, not grouped in clusters by TRIBE-MCL. (XLS 2 MB)

Additional file 4: **Occurrence of protein clusters, InterPro domains, molecular function GO terms and biological process GO terms in phylogenetic and lifestyle groups.** Protein clusters (A, B), InterPro domains (C, D), molecular function GO terms (E, F) and biological process GO terms (G, H) that have at least one member protein in each species of various defined phylogenetic (A, C, E, G) or lifestyle (B, D, F, H) groups but none in species outside these groups. (XLS 188 KB)

Additional file 5: **Contractions and expansions in protein clusters and InterPro term abundances in phylogenetic and lifestyle groups.** Protein clusters (A, B), InterPro domains (C, D), molecular function GO terms (E, F) and biological process GO terms (G, H) with contractions or expansions exclusively in each species of various defined phylogenetic (A, C, E, G) or lifestyle (B, D, F, H) groups, irrespective of contractions or expansions in species outside these groups. (XLS 188 KB)

Additional file 6: **Protein clusters showing non-universal contractions or expansions.** Selected protein clusters with contractions or expansions in some but not all species within specific phylogenetic lineages, irrespective of contractions or expansions in species in other phylogenetic lineages. (XLS 32 KB)

Additional file 7: **Protein cluster expansions in individual species.** Protein clusters having at least ten proteins and at least 50% more proteins in one species than in other species sharing this protein cluster. (XLS 37 KB)

Additional file 8: **InterPro domains and molecular function and biological process GO terms showing non-universal contractions and expansions in abundance in at least two but not all species within specific phylogenetic lineages, irrespective of contractions or expansions in species in other phylogenetic lineages.** (A) InterPro domain, molecular function and biological process GO terms that were identified in members from multiple protein clusters, with no protein cluster providing at least 75% of the total occurrences of that term. Terms highlighted in grey are described in Results. (B) InterPro domain, molecular function and biological process GO terms that were exclusively identified in members from the single accompanying protein clusters. (C) InterPro domain, molecular function and biological process GO terms, at least 75% of all occurrences of which were identified in members from the single accompanying protein clusters. (XLS 155 KB)

Additional file 9: **Numbers of Glycoside Hydrolases in predicted secretomes.** Glycoside Hydrolases were identified in predicted secretomes by comparison to the CAZy database using dbCAN. (XLS 176 KB)

Additional file 10: **InterPro domain, molecular function and biological process GO term abundance expansions in individual species.** Terms having at least ten occurrences and at least 50% more occurrences in one species than in other species sharing this term with corresponding protein clusters. (XLS 74 KB)
